# Bovine Tuberculosis: The Emergence of a New Wildlife Maintenance Host in Ireland

**DOI:** 10.3389/fvets.2021.632525

**Published:** 2021-03-25

**Authors:** David J. Kelly, Enda Mullen, Margaret Good

**Affiliations:** ^1^Discipline of Zoology, School of Natural Sciences, Trinity College Dublin, The University of Dublin, Dublin, Ireland; ^2^National Parks and Wildlife Service, Department of Housing, Local Government and Heritage, Dublin, Ireland; ^3^Independent Researcher and Private Consultant, Dun Laoghaire, Co. Dublin, Ireland

**Keywords:** Sika deer, Cervus nippon, tuberculosis, TB, cattle, European badger, maintenance host, Ireland

## Abstract

Despite advances in herd management, tuberculosis (TB) continues to affect ~0. 5% of Ireland's national cattle herd annually. It is clear that any “final” eradication of TB in cattle will need to address all TB maintenance hosts in the same environment. In Ireland and the UK, European Badgers (Meles meles) are a known TB maintenance host, while deer are recognised as spillover hosts. However, deer have been identified as maintenance hosts in other countries and Sika deer, specifically, have been identified with TB in Ireland. We examined the power of cattle, badger and Sika deer densities (at the county level) to predict cattle TB-breakdowns in Ireland, at both the herd and the individual level, using data collected between 2000 and 2018. Our hypothesis was that any positive correlations between deer density and cattle TB-breakdowns would implicate deer as TB maintenance hosts. Using linear multiple regressions, we found positive correlations between deer density and cattle TB-breakdowns at both the herd and individual levels. Since Sika deer in County Wicklow are known to have TB, we ran further regressions against subsets of data which excluded individual Irish counties. Analyses excluding Wicklow data showed much weaker correlations between Sika deer density and cattle TB-breakdowns at both the herd and individual levels, suggesting that these correlations are strongest in County Wicklow. A similar effect for badger density was seen in County Leitrim. While locally high densities of Sika deer persist in Irish counties, we believe they should be considered an integral part of any TB-control programme for those areas.

## Introduction

Tuberculosis (TB), an infectious disease caused by members of the *Mycobacterium tuberculosis* (*M. tuberculosis*) complex (MTBC) (1), is one of the leading causes of infectious disease mortality worldwide. As a zoonotic disease TB affects humans and multiple animal species and has been recognised as a major health risk to humans and animals for more than a century (2). In 2019, 1.4 million people died of tuberculosis (1). Bovine tuberculosis (bTB) is a chronic disease of cattle caused by members of the MTBC, primarily by *M. bovis*, but also *M. caprae* and, to a lesser extent, *M. tuberculosis* (3). In addition to infecting cattle and humans, the same Mycobacteria also infect other domestic animals and wildlife populations (4), causing general illness, pneumonia, weight loss and deaths. In countries where bTB is still common, and particularly where pasteurization of milk is not practiced, an estimated 10–15% of human cases of TB are caused by *M. bovis*.

Host density is a key driver of tuberculosis transmission rates and the aggregation of hosts (e.g., in large social groups) can create, or increase, opportunities for intra- or interspecific disease transmission (5). In Ireland bTB is most frequently caused by *M. bovis* (6). In cattle, TB transmission is most likely when stocking density is high (7). Depending on the infection dynamics of populations, infected wild animals are described as either maintenance or spillover hosts (8). In maintenance hosts, infection persists by intra-species transmission. By contrast, in spillover hosts, infection will not persist indefinitely, unless there is re-infection from another species.

The social structure of European Badgers (*Meles meles*) and their longevity, when infected, make them an ideal maintenance host for *M. bovis* (9). Indeed, badgers are recognised as wildlife reservoirs of *M. bovis* in the UK and Ireland (10). Other species are recognised as TB maintenance hosts in other countries; wild boar (*Sus scrofa*) in Iberia (11), African buffalo (*Syncerus caffer*) and Marsh antelope (*Kobus leche*) in Africa (12), Brushtail Possum (*Trichosurus vulpecula*) in New Zealand (13) and White-tailed Deer (*Odocoileus virginianus*) in the USA (14). In all cases, consideration of *M. bovis* (or other MTBC members) within these maintenance host populations is essential for bTB control programmes (15–19).

In general terms, disease emergence, or increased disease risk, is a frequent consequence of high density, close association and/or ungulate overabundance (20, 21). Red Deer (*Cervus elaphus*) and Fallow Deer (*Dama dama*), social species that naturally aggregate into groups, are considered TB spillover hosts in Spain (22), but recent work has shown that Red Deer, in Austria, Germany, and Italy, can also act as maintenance hosts for both *M. bovis* and *M. caprae* (another MTBC member) when they reach high densities (23–25). While regional density estimates appeared relatively low (e.g., 5.6 animals/km^2^), aggregation behaviour of Red Deer was shown to increase local densities by an order of magnitude (up to 46.2 animals/km^2^) at sites where supplementary winter feeding was provided (23). In the USA, White-tailed Deer numbers fluctuate from year to year in Michigan. In 1995 the population was estimated to be 2.2 million (26). Since that time the Michigan Department of Natural Resources (MDNR) have restricted supplementary winter feeding (27) and have attempted to reduce the state population to one million deer (in the spring herd). The MDNR have not been issuing estimates of deer populations for many years, but as the number of hunters and the deer harvest have both been on general declines since 1998 (28), we believe the White-tailed Deer population in Michigan is currently increasing. State-wide estimates of White-tailed Deer range from 4.5 to 8.8 animals/km^2^, using population estimates from 1937 to 1995, respectively (26). Yet the density of White-tailed Deer at DeSoto National Wildlife Refuge, Michigan, USA has been recorded between 36.5 and 50.6 animals/km^2^ (29). It is clear that aggregation behaviour in White-tailed Deer produces dramatic increases in local population density, in comparison to regional density estimates.

Estimates of Sika Deer (*Cervus nippon*) density for County Wicklow have risen from 7.8 animals/km^2^ in 2000 to 31.4 animals/km^2^ in 2018 (based on Sika Deer harvest or “bag” numbers published by the National Parks and Wildlife Service – NPWS–Supplementary Table 1). Although density calculations based on “bag” numbers are likely to be underestimates, as not all deer deaths (e.g., road deaths and natural causes) are reported in any given year, the technique has proved to be a good predictor of deer populations (30). The most recent estimate (31.4 animals/km^2^ in 2018) for Sika in County Wicklow provides a regional density far greater than that of both Red Deer in an Austrian bTB hotspot (23) and that of state-wide estimates for White-tailed Deer in Michigan (26). If Sika Deer exhibit aggregation behaviour similar to Red and White-tailed Deer, it is likely that they have reached a threshold density in County Wicklow and are now acting as maintenance hosts for TB, rather than spillover hosts.

TB has been recorded in both farmed and wild deer in Ireland (31), but records from the Irish Department of Agriculture, Food and the Marine (DAFM) show that Wicklow is the only county in Ireland where TB has been confirmed in multiple hunted deer over many years (6). In 2007/08, within Wicklow, 80 Sika deer were culled and their entire carcases examined for the presence of typical TB lesions. Incidence of TB was 5% and the strains of TB in the deer were also found to be present in local badger and cattle populations (6). In 2014/15 a more thorough TB detection protocol identified 17% prevalence (23 of 133 deer) (6). Unfortunately, it is unclear whether the difference in prevalence between these two studies represents a rise in TB infection in Wicklow's Sika Deer, as detection protocols varied between years. Despite that uncertainty, it is clear that TB has persisted in Wicklow's Sika deer for at least the last decade. A recent study (32) has identified a high level of diversity of TB strains in Sika deer in County Wicklow. The same study also found that the TB strains in the deer were shared between badgers and cattle (32). This indicates that deer are sharing TB with other TB hosts in their environment and may, therefore, be acting as a source of infection for local cattle populations. Thus, we investigated whether Sika deer met the characteristics of a wildlife reservoir host for *M. bovis* (15, 19).

## Materials and Methods

We conducted two multiple linear regression analyses. The first analysis (herd-level analysis) used the density of cattle herds in each Irish county experiencing bTB breakdowns as the dependent variable and densities of cattle herds, Sika deer and badgers in each Irish county, as well as the year of recording (2000–2018), as predictive variables. The second analysis (individual-level analysis) used the density of cattle which were removed under the bTB eradication programme (i.e., number of “reactors”) in each Irish county as the dependent variable, and densities of cattle, Sika deer, and badgers in each Irish county, as well as the year of recording, as predictive variables. The way in which the values for the dependent and predictive variables were determined is explained below.

### Estimations of Deer Populations From Hunting Bags

A study of Sika Deer in the Wicklow Mountains National Park (WMNP) (33) identified a nett annual productivity of about 25% (including estimations of female productivity, double births, calf survival, and adult survival). Another estimate (34) puts the nett annual productivity of Sika Deer in County Wicklow at 28%. This means that in order to maintain stable numbers, the maximum sustainable yield of local Sika Deer would be 28% of the population.

Using hunting bag data from the NPWS (www.npws.ie), we considered any increases in county bag numbers in consecutive years as an indication of population increase, i.e., more deer were being born than being removed. In those circumstances, bag totals were considered to be 25% of the population. When consecutive years showed slight decreases in hunting bags, those bag numbers were considered to indicate more deer were being removed than being born. We assumed those bags to represent 33% of the deer population. While bag numbers appear to offer a relatively crude method of assessing deer populations (35, 36), they can provide good estimates of county-level populations (30) (Supplementary Table 2). A variety of Sika density estimates were produced for each county by dividing population estimates of each deer species by habitat areas derived from CORINE 2012 (37) (Supplementary Table 3). This technique provided a way of allowing for deer aggregations in preferred habitats (e.g., pasture and woodland). The Sika density estimates which best explained variation in the herd-level (Supplementary Table 4) and individual-level (Supplementary Table 5) breakdown data for cattle were selected for those models, respectively, although all Sika density estimates gave similar results (Supplementary Tables 4, 5).

### Badger Population Estimates

A survey of badger main setts in Ireland (38) was used to provide a baseline for badger densities across Ireland. Despite attempts at modelling fluctuations in badger populations using occupancy rates (39), nett productivity estimates (40) and removals over the study period, we were unable to produce credible population estimates. In County Wicklow, a seven-year study of badgers in an unculled population, found that the local density remained stable between 2010 and 2016, despite numerous badgers dying on roads in the study area (41). Indeed, this population showed a nett migration into surrounding areas, where DAFM culling was in operation. If such a pattern were repeated across the country, it seems likely that culled areas would show only temporary reductions in local population density. Bearing this in mind, we assumed that badger populations, at the county level, remained constant over the study period (Supplementary Table 6). This assumption tallies with anecdotal data.

A variety of badger density estimates were produced for each county by dividing estimates of badger numbers by habitat areas derived from CORINE 2012 (37). The badger density estimates which best explained variation in the herd-level (Supplementary Table 7) and individual level (Supplementary Table 8) breakdown data for cattle were selected for those models, respectively. As the badger populations in all counties were assumed to be stable, badger density values for individual counties were constant across the study period (2000–2018) in both the herd-level and individual-level models.

### Cattle Population Densities

We used data from DAFM to identify the number of herds registered in each county (Supplementary Table 9), the number of infected herds within each county (Supplementary Table 10), the number of cattle removed as reactors in each county (Supplementary Table 11) and the number of individual cattle in each county (Supplementary Table 12), for each year of the study period (42).

Density estimates of herds, or individuals, were produced by dividing cattle numbers for each county by pasture areas derived from CORINE 2012 (37) (Supplementary Table 3).

### Statistical Analysis

Analyses were performed in R (43) we used the *lmer* function from the **lme4** package (44) to perform Generalised Linear Models (GLMs). Data were centred and scaled, to remove any numeric bias from individual predictive variables, using the *standardize* function from the **arm** package (45). County cattle density, county badger density, county deer density, year and all two-way interactions of animal densities with year, were included in full models for herd-level and individual-level analyses. The R code for these models is included for the reference of readers (Supplementary Figure 1).

## Results

### Herd-level Analysis

This analysis used the density of cattle herds, Sika deer and badgers to explain the variation in herds with breakdowns at the county level between 2000 and 2018. Cattle herd densities were calculated per square kilometre of pasture. Following a comparison of density alternatives which best explained the variation in herd TB-breakdown density (Supplementary Table 4), Sika deer densities were calculated per combined square kilometre of pasture and forestry. A similar comparison for badgers (Supplementary Table 7), identified densities calculated per square kilometre of pasture as most appropriate.

The GLM provided several details about the trends in herd breakdown density (Table 1). Over the period of the study (2000–2018), the number of herd breakdowns fell (Year; estimate = −0.547431, *t* = −19.93, *P* < 0.001). The density of cattle herds was strongly and positively correlated with herd breakdown density (Cattle Herds; estimate = 0.319594, *t* = 6.877, *P* < 0.001), and this correlation showed a slight (but statistically insignificant) weakening over the course of the study (Cattle:Year interaction; estimate = −0.114097, *t* = −1.259, *P* > 0.05). The density of badgers was also strongly and positively correlated with the herd breakdown density (Badgers; estimate = 0.234398, *t* = 5.201, *P* < 0.001), and this correlation also weakened over the course of the study (Badger:Year interaction; estimate = −0.222166, *t* = −2.487, *P* = 0.0132). The density of Sika deer was weakly (lacking statistical significance) positively correlated with herd breakdown density (Deer; estimate = 0.05551, *t* = 1.674, *P* > 0.05), but the strength of this correlation increased over the course of the study (Deer:Year interaction; estimate = 0.139537, *t* = 2.134, *P* = 0.0334).

**Table 1:** Output from the GLM of herd-level analysis.

	**Estimate**	**Std. Error**	***t*-value**	**Pr(>|t|)**	**Significance**
(Intercept)	−0.005468	0.01371	−0.399	0.6902	
Cattle (herd) density	0.319594	0.046474	6.877	<0.001	^***^
Badger density	0.234398	0.045066	5.201	<0.001	^***^
Deer density	0.05551	0.033163	1.674	0.0948	
Year	−0.547431	0.027464	−19.93	<0.001	^***^
Cattle: Year	−0.114097	0.090601	−1.259	0.2085	
Badger: Year	−0.222166	0.089342	−2.487	0.0132	^*^
Deer: Year	0.139537	0.065393	2.134	0.0334	^*^

The nature of the correlations between explanatory variables and the dependent variable (cattle herd breakdown density) are indicated by the values in the Estimate column; positive values indicate a positive correlation and negative values indicate a negative correlation. The P-values of correlations are given in the Pr(>|t|) column, and the starred rating of these correlations is given in the significance column;

* *p < 0.05*,

** *p < 0.01*,

*** *p < 0.001*.

### Individual-level Analysis

This analysis used the density of cattle, Sika deer and badgers to explain the variation in the density of “reactor” cattle from herds with breakdowns, at the county level, between 2000 and 2018. Cattle densities were calculated per square kilometre of pasture. Following a comparison of density alternatives which best explained the variation in “reactor” density (Supplementary Table 5), Sika deer densities were calculated per square kilometre of agricultural land. A similar comparison for badgers (Supplementary Table 8), identified densities calculated per square kilometre of pasture as most appropriate.

The GLM provided several details about the trends in “reactor” cattle density (Table 2). Over the period of the study (2000–2018), the number of “reactors” fell (Year; estimate = −0.43572, *t* = −12.582, *P* < 0.001). The density of cattle was strongly and positively correlated with “reactor” density (Cattle; estimate = 0.2498, *t* = 4.831, *P* < 0.001), but the strength of this correlation showed a dramatic reduction over the course of the study (Cattle:Year interaction; estimate = −0.39112, *t* = −4.095, *P* < 0.001). The density of badgers was positively correlated with “reactor” breakdown density (Badgers; estimate = 0.15049, *t* = 2.983, *P* = 0.03), but this correlation did not change over the course of the study (Badger:Year interaction; estimate = 0.03452, *t* = 2.983, *P* > 0.05). The density of Sika deer was strongly and positively correlated with “reactor” density (Deer; estimate = 0.18767, *t* = 4.461, *P* < 0.001). While the strength of this correlation increased over the course of the study, that change did not reach statistical significance (Deer:Year interaction; estimate = 0.13961, *t* = 1.712, *P* = 0.0876).

**Table 2:** Output from the GLM of individual-level analysis.

	**Estimate**	**Std. Error**	***t*-value**	**Pr(>|t|)**	**Significance**
(Intercept)	−0.01777	0.01719	−1.034	0.3016	
Cattle (“reactor”) density	0.24980	0.05170	4.831	1.82 e−06	^***^
Badger density	0.15049	0.05045	2.983	0.0030	^**^
Deer density	0.18767	0.04207	4.461	1.01 e−05	^***^
Year	−0.43572	0.03463	−12.582	2.00 e−16	^***^
Cattle: Year	−0.39112	0.09552	−4.095	4.95 e−05	^***^
Badger: Year	0.03452	0.09598	0.360	0.7193	
Deer: Year	0.13961	0.8157	1.712	0.0876	

The nature of the correlations between explanatory variables and the dependent variable (cattle “reactor” density) are indicated by the values in the Estimate column; positive values indicate a positive correlation and negative values indicate a negative correlation. The P-values of correlations are given in the Pr(>|t|) column, and the starred rating of these correlations is given in the significance column;

* *p < 0.05*,

** *p < 0.01*,

*** *p < 0.001*.

### General Findings

Although there were differences between the two analyses, some general effects can be seen. Principally, the incidence of TB in cattle reduced dramatically during the study period. Despite this reduction, the local density of cattle, badgers, and Sika deer were all positively correlated with local TB density. While the correlation between local cattle density and local TB incidence, along with the correlation between local badger density with local TB incidence have been decreasing over time, the correlation between local deer density and local TB incidence has been increasing.

Having established that local Sika deer density was a useful predictor of local TB incidence, we investigated whether there were any regional aspects to this relationship. We ran further iterations of the herd-level and individual level analyses with subsets of the national data, excluding individual counties.

When Wicklow data were removed from the herd-level dataset (Supplementary Table 13), the resultant model no longer identified Sika deer density as an important predictor of TB breakdowns (Deer:Year interaction; estimate = 0.030361, *t* = 0.431, *P* = 0.6665). The same trend was seen when Westmeath data were removed from the herd-level dataset (Supplementary Table 13) (Deer:Year interaction; estimate = 0.130046, *t* = 1.955, *P* = 0.0512).

When Wicklow data were removed from the individual-level dataset (Supplementary Table 14), the resultant model no longer identified Sika deer density as an important predictor of TB reactors (Deer; estimate = −0.05224, *t* = −1.258, *P* = 0.20896). County Wicklow was the only county which affected the individual-level analysis in this way.

Curiously, when Leitrim data were removed from the herd-level dataset (Supplementary Table 13), the resultant model no longer identified badger density as an important predictor of TB breakdowns (Badgers; estimate = 0.040442, *t* = 1.203, *P* = 0.2295). The same trend was seen when Leitrim data were removed from the individual-level dataset (Supplementary Table 14) (Badgers; estimate = 0.05352, *t* = 1.316, *P* = 0.1887).

## Discussion

### Herd-level Analysis

Higher local cattle densities and higher local badger densities correlate with higher local cattle herd TB-breakdown densities (Table 1). Such correlations are expected, since cattle and badgers are known maintenance hosts of TB in Ireland (46). The herd-level model shows the correlation between local badger density and local cattle herd TB-breakdown density has been decreasing over the last 19 years. This suggests that the national bTB eradication policy in Ireland (47, 48), and in particular the control of TB in the badger population, has been achieving success. Indeed, recent monitoring of TB in culled badgers has shown a reduction in infection rate from 26% in 2007 to 11% in 2011 (49). Such progress may encourage a progressive shift from culling towards badger vaccination (50). However, the correlation between local cattle herd density and local cattle herd TB-breakdown density has remained static. This indicates that whatever controls on herds are currently in place have been insufficient to make significant reductions in herd-level TB infection over the last 19 years. A recent study has suggested that high risk herds should be monitored even more closely (50).

While the management of cattle and badgers appears to have reduced local rates of TB breakdowns in cattle herds, Sika populations do not appear to have been under management. Increases in local Sika deer densities were correlated with an increase in local rates of TB breakdowns in cattle herds (Table 1). From further analyses it appears that the data from Counties Wicklow and Westmeath were driving this correlation (Supplementary Table 13). These counties show the highest correlations of Sika deer density with TB levels in cattle. It is here that we believe Sika deer, as maintenance hosts of TB, pose the greatest threat to cattle.

### Individual-level Analysis

Higher local cattle densities and higher local badger densities correlate with higher numbers of “reactor” cattle at test (Table 2). This is to be expected, as cattle and badgers are known maintenance hosts of TB in Ireland (46). Our individual-level model identifies the correlation between local cattle density and local “reactor” density has decreased over the study period. This suggests that herd management and monitoring practices have improved over the last 19 years. Potentially, it is these improvements which have prevented the influence of TB wildlife vectors (i.e., badgers or deer) changing over the course of the study. However, the local density of Sika deer was strongly positively correlated with the density of “reactor” cattle. It appears that data from County Wicklow were driving this correlation (Supplementary Table 14).

### Sika Deer as Maintenance Hosts of *M. bovis*

While a growing body of evidence (6, 32) has identified TB in Sika deer in Wicklow, it has been difficult to identify Sika deer as maintenance hosts of TB. Several unique strains of *M. bovis* have been found in Sika deer within Wicklow (32), which suggests they act as wildlife reservoirs of TB. The data presented here provides clear evidence that higher levels of TB in cattle are associated with higher local densities of Sika deer. While this does not demonstrate Sika deer act as maintenance hosts of TB in Ireland, it adds further weight to the argument.

Sika deer in Wicklow now have regional densities comparable to other deer populations where TB maintenance has been demonstrated (23, 51). While no other deer species are implicated in our findings, Roe deer (*Capreolus capreolus*) are known to act as spillover hosts (52), and both Red deer (10, 23) and Fallow deer (53, 54) have been found to act as TB maintenance hosts at higher densities. So, while we would encourage active management of Sika Deer in Wicklow, we would also encourage the monitoring of Sika, Red and Fallow deer numbers in other counties. Such monitoring should be of particular interest to those who harbour the ambition of making Ireland TB-free by 2030 (48), or England TB-free by 2038 (55), as well as stakeholders in countries where the management of bovine TB continues to cost tax-payers eye-watering sums (48, 55).

It is difficult to offer clear guidelines regarding a “safe” Sika deer population density within Wicklow. Sika are not distributed evenly across the county (Wesley Atkinson *pers. comm*.), so assessment of density may require calculation at a finer scale (e.g., electoral district). Normal aggregation behaviour by deer means that local population densities may be an order of magnitude higher than the overall regional population densities (23). While we are unaware of a tradition of supplementary feeding of wild deer in Ireland, White-tailed deer in the United States (56) and Red deer in the Tyrol (23) may be encouraged to aggregate when they receive supplementary winter feed. The practice of providing supplementary feeding at pasture to farmed livestock grazing, in the vicinity of Sika habitat, may offer unintended supplementary feed for deer. This could promote aggregation behaviour and bring deer and cattle into contact at high density. Allowing cattle access to woodland, or higher rough-grazing areas, would also increase cattle-deer interaction opportunities, as well as potentially increasing MTBC contamination in the environment where persistence of these diseases and exposure to susceptible species is a concern (4). Interspecies transmission of MTBC has been reported at interface areas between species (57–59).

Our findings serve as a timely reminder that the final eradication of TB in any national cattle herd, is likely to prove problematic unless all MTBC diseases are addressed in all livestock (60) and wildlife reservoirs (4, 16–19, 61).

## Data Availability Statement

The original contributions presented in the study are included in the article/supplementary material, further inquiries can be directed to the corresponding authors.

## Ethics Statement

Ethical review and approval was not required for the animal study because while the species considered in this study were vertebrates, we were not required to conduct any experiments with them. We relied on data which had already been collected and was freely available in the public domain.

## Author Contributions

DK formulated the idea, which was then developed by DK, MG, and EM. MG and EM sourced the data. DK developed the models to analyse the data and drafted the manuscript. MG fleshed out the outline. All authors continually reviewed and edited the manuscript, in order to produce the submitted draft.

## Conflict of Interest

The authors declare that the research was conducted in the absence of any commercial or financial relationships that could be construed as a potential conflict of interest.

## References

[1] WHO. Global Tuberculosis Report. Geneva: World Health Organisation (2020).

[2] Olea-Popelka F Muwonge A Perera A Dean AS Mumford E Erlacher-Vindel E. Zoonotic tuberculosis in human beings caused by Mycobacterium bovis-a call for action. Lancet Infect Dis. (2017) 17:E21–5. 10.1016/S1473-3099(16)30139-6 27697390

[3] OIE. Terrestrial Animal Health Code Chapter 8.11. P: Office International des Epizooties(2019).

[4] Kanipe C Palmer MV. *Mycobacterium bovis* and you: a comprehensive look at the bacteria, its similarities to *Mycobacterium tuberculosis*, and its relationship with human disease. Tuberculosis. (2020) 125:102006. 10.1016/j.tube.2020.102006 33032093

[5] Estrada-Pena A Ostfeld RS Peterson AT Poulin R de la Fuente J. Effects of environmental change on zoonotic disease risk: an ecological primer. Trends Parasitol. (2014) 30:205–14. 10.1016/j.pt.2014.02.003 24636356

[6] Good M Duignan A. Veterinary handbook for herd management in the bovine TB eradication programme. Department of Agriculture, Food and the Marine, Dublin, Ireland (2017).

[7] Neill SD Hanna J Obrien JJ McCracken RM. Transmission of tuberculosis from experimentally infected cattle to in-contact calves. Vet Rec. (1989) 124:269–71. 10.1136/vr.124.11.269 2652875

[8] Morris RS Pfeiffer DU. Directions and issues in bovine tuberculosis epidemiology and control in New Zealand. N Z Vet J. (1995) 43:256–65. 10.1080/00480169./1995.35904 16031864

[9] Gormley E Corner LAL. Pathogenesis of *Mycobacterium bovis* infection: the badger model as a paradigm for understanding tuberculosis in animals. Front Vet Sci. (2018) 4:247. 10.3389/fvets.2017.00247 29379792 PMC5775213

[10] Gortázar C Delahay RJ McDonald RA Boadella M Wilson GJ Gavier-Widén D. The status of tuberculosis in European wild mammals. Mammal Rev. (2012) 42:193–206. 10.1111/j.1365-2907.2011.00191.x

[11] Muñoz-Mendoza M Marreros N Boadella M Gortázar C Menéndez S de Juan L. Wild boar tuberculosis in Iberian Atlantic Spain: a different picture from Mediterranean habitats. BMC Vet Res. (2013) 9:176. 10.1186/1746-6148-9-176 24010539 PMC3844463

[12] Renwick AR White PCL Bengis RG. Bovine tuberculosis in southern African wildlife: a multi-species host-pathogen system. Epidemiol Infect. (2007) 135:529–40. 10.1017/S0950268806007205 16959052 PMC2870607

[13] Nugent G Buddle BM Knowles G. Epidemiology and control of *Mycobacterium bovis* infection in brushtail possums (*Trichosurus vulpecula*), the primary wildlife host of bovine tuberculosis in New Zealand. N Z Vet J. (2015) 63:28–41. 10.1080/00480169.2014.963791 25290902 PMC4566891

[14] VerCauteren KC Lavelle MJ Campa H. Persistent spillback of bovine tuberculosis from white-tailed deer to cattle in Michigan, USA: Status, strategies, and needs. Front Vet Sci. (2018) 5:301. 10.3389/fvets.2018.00301 30555834 PMC6281989

[15] Corner LAL. The role of wild animal populations in the epidemiology of tuberculosis in domestic animals: how to assess the risk. Vet Microbiol. (2006) 112:303–12. 10.1016/j.vetmic.2005.11.015 16326039

[16] Francis J. Tuberculosis in Animals and Man: a Study in Comparative Pathology. London: Cassell & Co. Ltd (1958).

[17] Good M Bakker D Duignan A Collins DM. The history of *in vivo* tuberculin testing in bovines: tuberculosis, a “One Health” issue. Front Vet Sci. (2018) 5:59. 10.3389/fvets.2018.00059 29686992 PMC5900347

[18] Martinez-Guijosa J Romero B Infantes-Lorenzo JA Diez E Boadella M Balseiro A. Environmental DNA: a promising factor for tuberculosis risk assessment in multi-host settings. PLoS ONE. (2020) 15:e0233837. 10.1371/journal.pone.0233837 32470035 PMC7259669

[19] Palmer MV. *Mycobacterium bovis*: characteristics of wildlife reservoir hosts. Transbound Emerg Dis. (2013) 60:1–13. 10.1111/tbed.12115 24171844

[20] Gortázar C Acevedo P Ruiz-Fons F Vicente J. Disease risks and overabundance of game species. Eur J Wildl Res. (2006) 52:81–7. 10.1007/s10344-005-0022-2

[21] Ryan TJ Livingstone PG Ramsey DSL de Lisle GW Nugent G Collins DM. Advances in understanding disease epidemiology and implications for control and eradication of tuberculosis in livestock: the experience from New Zealand. Vet Microbiol. (2006) 112:211–9. 10.1016/j.vetmic.2005.11.025 16330161

[22] Aranaz A de Juan L Montero N Sanchez C Galka M Delso C. Bovine tuberculosis (*Mycobacterium bovis*) in wildlife in Spain. J Clin Microbiol. (2004) 42:2602–8. 10.1128/JCM.42.6.2602-2608.2004 15184440 PMC427808

[23] Fink M Schleicher C Gonano M Prodinger WM Pacciarini M Glawischnig W Ryser-Degiorgis MP. Red deer as maintenance host for bovine tuberculosis, Alpine Region. Emerg Infect Dis. (2015) 21:464–7. 10.3201/eid2103.141119 25695273 PMC4344270

[24] Kohl TA Utpatel C Nieman S Moser I. Mycobacterium bovis persistence in two different captive wild animal populations in Germany: a longitudinal molecular epidemiological study revealing pathogen transmission by whole-genome sequencing. J Clin Microbiol. (2018) 56:e00302–18. 10.1128/JCM.00302-18 29950330 PMC6113487

[25] Schoepf K Prodinger WM Glawischnig W Hofer E Revilla-Fernandez S Hofrichter J. A two-years' survey on the prevalence of tuberculosis caused by *Mycobacterium caprae* in red deer (*Cervus elaphus*) in the Tyrol, Austria. ISRN Vet Sci. (2012) 2012:245138. 10.5402/2012/245138 23762580 PMC3671721

[26] MDNR. Michigan deer management plan. Michigan Department of Natural Resources, Lansing, Michigan, USA (2016).

[27] Cosgrove MK O'Brien DJ Ramsey DSL. Baiting and feeding revisited: Modeling factors influencing transmission of tuberculosis among deer and to cattle. Front Vet Sci. (2018) 5:306. 10.3389/fvets.2018.00306 30564585 PMC6288431

[28] Frawley BJ. Michigan Deer Harvest Survey Report 2018 Seasons. Michigan Department of Natural Resources (2019).

[29] Hefley TJ Hygnstrom SE Gilsdorf JM Clements GM Clements MJ Tyre AJ. Effects of deer density and land use on mass of white-tailed deer. J Fish Wildl Manag. (2013) 4:20–32. 10.3996/022012-JFWM-015

[30] Adams HL Kissell RE Ratajczak D Warr EL Applegate RD Barrett L. Relationships among white-tailed deer density, harvest, and landscape metrics in TN, USA. Eur J Wildl Res. (2020) 66:19. 10.1007/s10344-019-1353-8

[31] Quigley FC Costello E Flynn O Gogarty A McGuirk J Murphy A. Isolation of mycobacteria from lymph node lesions in deer. Vet Rec. (1997) 141:516–8. 10.1136/vr.141.20.516 9416676

[32] Crispell J Cassidy S Kenny K McGrath G Warde S Cameron H. *Mycobacterium bovis* genomics reveals transmission of infection between cattle and deer in Ireland. Microb Genomics. (2020) 6:mgen000388. 10.1099/mgen.0.000388 32553050 PMC7641417

[33] Atkinson W. To Assess the Productivity, Condition, and Reproductive Success of Deer in the Wicklow Mountains National Park From Post-Mortem Cull Data Between 1995 and 1998. Diploma in Field Ecology, University College Cork (1998).

[34] O'Brien D Rooney SM Hayden TJ. Reproduction and potential rate of increase of the sika deer herd in County Wicklow. Ir For. (2007) 64:32–43.

[35] Borchers DL Buckland ST Zucchini W. Estimating Animal Abundance. London: Springer (2002). 10.1007/978-1-4471-3708-5

[36] Morellet N Klein F Solberg E Andersen R The census and management of populations of ungulates in Europe. In: Putman RJ Apollonio M Andersen R editors. Ungulate Management in Europe-Problems and Practices. Cambridge: Cambridge University Press (2011). p. 106–43. 10.1017/CBO9780511974137.006

[37] Lydon K Smith G. CORINE landcover 2012 Ireland-Final Report. Environmental Protection Agency, Johnstown Castle, County Wexford, Ireland (2014)

[38] Smal C. A badger and habitat survey of Ireland. The Stationary Office, Dublin, Ireland (1995).

[39] Sleeman DP Davenport J More SJ Clegg TA Collins JD Martin SW. How many Eurasian badgers *Meles meles* L. are there in the Republic of Ireland? Eur J Wildl Res. (2009) 55:333–44. 10.1007/s10344-008-0244-1

[40] Anderson RM Trewhella W. Population-dynamics of the badger (*Meles meles*) and the epidemiology of bovine tuberculosis (*Mycobacterium bovis*). Philos Trans R Soc London Ser B. (1985) 310:327–81. 10.1098/rstb.1985.0123 2865760

[41] Gaughran A Kelly DJ MacWhite T Mullen E Maher P Good M. Super-ranging. A new ranging strategy in European badgers. PLoS ONE. (2018) 13:e0191818. 10.1371/journal.pone.0191818 29444100 PMC5812585

[42] DAFM. Bovine Tuberculosis by Regional Veterinary Offices, Year and Statistical Indicator. Central Statistics Office (2020).

[43] R Core Team R. A Language and Environment for Statistical Computing. Vienna: R Foundation for Statistical Computing (2020).

[44] Bates D Maechler M Bolker B Walker S. Fitting linear mixed-effects models using lme4. J Stat Softw. (2015) 67:1–48. 10.18637/jss.v067.i01

[45] Gelman A Su Y-S. arm: Data analysis using regression and multilevel/hierarchical models (2020). Available online at: https://CRAN.R-project.org/package=arm

[46] More SJ. Can bovine TB be eradicated from the Republic of Ireland? Could this be achieved by 2030? Ir Vet J. (2019) 72:3. 10.1186/s13620-019-0140-x 31057791 PMC6485114

[47] DAFM. National farmed animal health strategy. Department of Agriculture, Food and the Marine, Dublin, Ireland (2017)

[48] DAFM. Bovine TB stakeholder forum. Department of Agriculture, Food and the Marine, Dublin, Ireland (2018)

[49] Byrne AW Kenny K Fogarty U O'Keeffe JJ More SJ McGrath G. Spatial and temporal analyses of metrics of tuberculosis infection in badgers (Meles meles) from the Republic of Ireland: Trends in apparent prevalence. Prev Vet Med. (2015) 122:345–54. 10.1016/j.prevetmed.2015.10.013 26556049

[50] Houtsma E Clegg TA Good M More SJ. Further improvement in the control of bovine tuberculosis recurrence in Ireland. Vet Rec. (2018) 183:622. 10.1136/vr.104642 30171099 PMC6288696

[51] Palmer MV Thacker TC Waters WR Robbe-Austerman S. Oral vaccination of white-tailed deer (*Odocoileus virginianus*) with *Mycobacterium bovis* Bacillus Calmette-Guerin (BCG). PLoS ONE. (2014) 9:e97031. 10.1371/journal.pone.0097031 24804678 PMC4013142

[52] Lambert S Hars J Reveillaud E Moyen JL Gares H Rambaud T. Host status of wild roe deer in bovine tuberculosis endemic areas. Eur J Wildl Res. (2017) 63:15. 10.1007/s10344-016-1071-4

[53] Griffin JFT Chinn DN Rodgers CR. Diagnostic strategies and outcomes on three New Zealand deer farms with severe outbreaks of bovine tuberculosis. Tuberculosis. (2004) 84:293–302. 10.1016/j.tube.2003.11.001 15207804

[54] Robinson RC Phillips PH Stevens G Storm PA. An outbreak of *Mycobacterium bovis* infection in fallow deer (*Dama dama*). Aust Vet J. (1989) 66:195–7. 10.1111/j.1751-0813.1989.tb09806.x 2673180

[55] Defra. The strategy for achieving officially bovine tuberculosis free status for England. Department for Environment, Food and Rural Affairs, London, United Kingdom (2014). Available online at: www.gov.uk/defra

[56] Bowman B Belant JL Beyer DE Martel D. Characterizing nontarget species use at bait sites for white-tailed deer. Human Wildlife Interact. (2015) 9:110–8.

[57] Katale BZ Mbugi EV Siame KK Keyyu JD Kendall S Kazwala RR. Isolation and potential for transmission of *Mycobacterium bovis* at human-livestock-wildlife interface of the Serengeti Ecosystem, Northern Tanzania. Transbound Emerg Dis. (2017) 64:815–25. 10.1111/tbed.12445 26563417 PMC5434928

[58] LaHue NP Banos JV Acevedo P Gortazar C Martinez-Lopez B. Spatially explicit modeling of animal tuberculosis at the wildlife-livestock interface in Ciudad Real province, Spain. Prev Vet Med. (2016) 128:101–11. 10.1016/j.prevetmed.2016.04.011 27237396

[59] Sichewo PR Hlokwe TM Etter EMC Michel AL. Tracing cross species transmission of *Mycobacterium bovis* at the wildlife/livestock interface in South Africa. BMC Microbiol. (2020) 20:49. 10.1186/s12866-020-01736-4 32131736 PMC7057561

[60] Kean JM Barlow ND Hickling GJ. Evaluating potential sources of bovine tuberculosis infection in a New Zealand cattle herd. New Zeal J Agr Res. (1999) 42:101–6. 10.1080/00288233.1999.9513358

[61] Balseiro A Thomas J Gortázar C Risalde MA. Development and Challenges in Animal Tuberculosis Vaccination. Pathogens. (2020) 9:472. 10.3390/pathogens9060472 32549360 PMC7350370

